# Immunotherapy in Recurrent/Metastatic Squamous Cell Carcinoma of the Head and Neck

**DOI:** 10.3389/fonc.2021.705614

**Published:** 2021-09-01

**Authors:** Ronan W. Hsieh, Steven Borson, Anastasia Tsagianni, Dan P. Zandberg

**Affiliations:** Division of Hematology/Oncology, UPMC Hillman Cancer Center, Pittsburgh, PA, United States

**Keywords:** head and neck cancer, HNSCC, recurrent, metastatic, systemic therapy, immunotherapy, PD-1, PD-L1

## Abstract

Head and neck cancer is the 6^th^ most common cancer worldwide with the most common histology being squamous cell carcinoma (HNSCC). While the majority of patients present at a stage where curative intent therapy is possible, when patients recur and/or develop metastatic disease, outcomes are generally poor, especially with systemic therapy alone, and they lag behind other solid tumors. Over the last decade immunotherapy has revolutionized the field of oncology, and anti-PD-1-based therapy has changed the standard of care in recurrent/metastatic (R/M) HNSCC as well. With these gains have come new questions to continue to move the field forward. In this review, we discuss the tumor immune microenvironment and predictive biomarkers and current status and future directions for immunotherapy in recurrent/metastatic head and neck cancer.

## Introduction

Head and neck cancer is the 6^th^ most common cancer worldwide, and while it includes many histologies, squamous cell carcinoma represents 90% of diagnosis, with the most common primary sites being oral cavity, hypopharynx, larynx, and oropharynx (1). In addition to traditional risk factors of smoking and alcohol, there are two virally driven cancers, the Epstein Barr Virus (EBV) in the nasopharynx and the Human Papillomavirus (HPV) in the oropharynx, with the latter associated with a significantly better prognosis (2). While the majority of squamous cell carcinoma of the head and neck (HNSCC) patients present at a stage where therapy is definitive, with only 10% presenting with distant metastatic disease, a large proportion of patients, especially HPV negative HNSCC, will recur. In the recurrent/metastatic (R/M) setting there is a great need for improvement in outcomes, especially when treatment is with systemic therapy alone. Immunotherapy has changed our standard-of-care approach and improved outcomes in this setting, but there is still more work to do to continue to move the needle forward. In this review we detail the current status of immunotherapy in R/M HNSCC, predictive biomarkers, and future directions in the field.

## The Tumor Immune Microenvironment in HNSCC

Antitumor immunity is a back-and-forth duel between the immune system and the cancer. The cancer immunoediting theory hypothesizes that at first the immune system recognizes and eliminates all cancer cells, then the cancer evades the immune system such that only equilibrium is achieved in which tumor growth is controlled but not eradicated, followed by the “escape” phase whereby the tumor fully eludes the immune system and progresses clinically (3). Numerous steps need to occur in order for the immune system to achieve effective cancer killing. The process begins with the release of cancer neoantigens and their uptake by antigen-presenting cells such as dendritic cells (DC) with subsequent required signaling to move forward with presentation on MHC I and MHC II molecules to T cells (4). Next, effector T cells are activated and then migrate to and infiltrate the tumor microenvironment (5, 6). Then finally T cells bind to the target cancer cells *via* their T cell receptors (TCR) and kill them *via* multiple mechanisms (7, 8). HNSCC, like other cancers, can evade or suppress the immune response at each of these steps. For example, in HNSCC, the tumor-infiltrating T cells can be compromised *via* functional defects leading to decreased proliferation in response to cytokines, impaired ability to kill tumor cells, and suppressed IL-1 and/or IFN-γ production (9–13). Moreover, the cytotoxic properties of NK cells are inhibited *via* TGF- *β*1 overexpression that leads to reduced expression of NK cell receptors KHG2D and CD16 (14). HNSCC can modulate the immune response to favor induction and conversion to immunosuppressive cells such as Tregs, which are abundant in the tumor microenvironment (TME) as well as peripheral blood, exerting their immunosuppressive function by inducing apoptosis of CD8+ T cells and inhibiting proliferation of CD4+ T cells (15–17). Additionally, Myeloid-Derived Suppressor Cells (MDSCs) can inactivate T cells *via* production of arginase-1 and inducible NO synthase (18, 19). Finally, stromal fibroblasts as well as non-cellular components of TME including growth factors, glycoproteins, and structural proteins produced by Extracellular Matrix (ECM) further enhance tumor invasion, migration, and progression (20–23).

Another important mechanism the tumor uses to modify the immune response and block antitumor immunity is *via* manipulation of co-signaling molecule signaling. Co-signaling molecules can be stimulatory or inhibitory on immune function. This includes the most studied and clinically relevant programmed cell death protein 1 (PD-1): Programmed death ligand 1 (PD-L1) pathway. PD-1 is a member of the CD28 family of T-cell costimulatory receptors and is expressed on activated T cells, B cells, and monocytes (24–26). In addition to tumor cells, PD-L1 is expressed on activated T cells, B cells, NK (natural killer) cells, dendritic cells, macrophages, and non-hematopoietic cells (27, 28). Importantly, PD-L1 can be upregulated in tumor cells *via* inflammatory signals, mainly under the influence of IFN-γ produced by immune cells and activation of downstream pathways such as EGFR, MAPK, or PI3K-Akt (29–34). Even before monoclonal antibodies made it into the clinic, PD-L1 expression was observed in HNSCC, ranging from 46 to 100% in primary, recurrent, and metastatic settings (34–40). The ligation of PD-1 by PD-L1 or PD-L2 suppresses antitumor response *via* effector T-cell exhaustion and/or apoptosis (26). In addition to the effector T cell tumor interface, antitumor immunity can be induced by blockade of the PD-L1:PD-1 pathway on dendritic cells, resulting in increased CD8-positive T cell infiltration of the tumor and suppression of the inhibitory ability of Tregs either directly or indirectly through augmentation of CTL proliferation (41, 42). Moreover, PD-L1 can ligate B7-1 (CD80), a costimulatory molecule found on T cells, that regulates the downstream immune responses through the PD-1 pathway (43). Other relevant inhibitory co-signaling molecules expressed in HNSCC that are already the target of therapeutic intervention include CTLA4, LAG3, B7-H3, TIGIT, TIM3, and stimulatory OX40, ICOS, GITR, and 4-1BB (44, 45).

**Figure 1:**
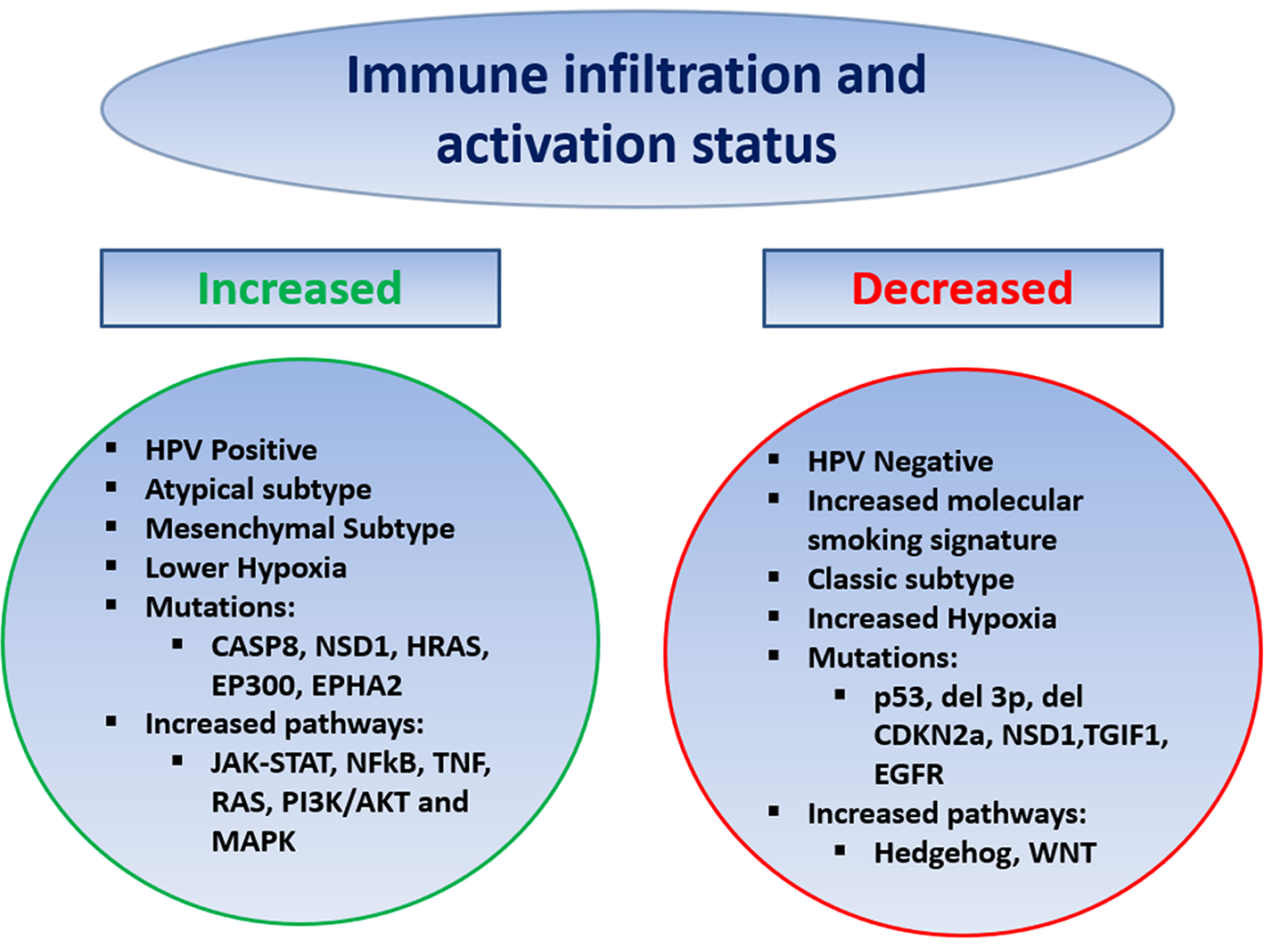
Predictors of the Tumor Immune Microenvironment in HNSCC.

In HNSCC the tumor immune microenvironment (TIME) has been analyzed *via* various methods ranging from immunohistochemistry to genomic and transcriptomic analysis, examining the effect of HPV, molecular smoking signatures, and other genomic predictors (**Figure 1**). Using bulk RNA sequencing data from The Cancer Genome Atlas (TCGA), HNSCC tumors showed high levels of immune infiltration, including NK cells, with the highest infiltration by Tregs and Treg/CD8 ratio, compared to nine other solid tumors including NSCLC, RCC, melanoma, and breast (46). Delineating a T cell inflamed phenotype (TCIP) using a validated chemokine gene expression signature, 34% of HNSCC tumors were characterized as high, 32% intermediate, and 34% low. TCIP high phenotype correlated with increased CD8 T cell infiltration and mesenchymal subtype but also increased exhaustion/cytotoxic CD8 T cell ratio and higher inhibitory co-signaling molecule expression of PD-L1, PD-1, CTLA4, TIM3, and LAG3 compared to TCIP-low. TCIP high tumors were enriched in pathways including JAK-STAT, NFkB, TNF, RAS, PI3K/AKT, and MAPK, whereas Hedgehog and WNT/B-catenin signaling was associated with TCIP low (47).

Multiple studies have compared the TIME by HPV status. Mandal and colleagues observed that HPV positive HNSCC was associated with a higher immune infiltrate and activation status by a cytolytic score, as well as increased Treg and Treg/CD8 ratio compared to HPV negative. Specifically, in regard to Tregs, other studies have similarly found an increase in HPV positive HNSCC, while other analysis have not shown a difference by HPV status (46, 48, 49). The TIME of HPV positive HNSCC has been observed to have increased NK cells, M1 macrophages (as compared to M2), and CD8 T cells, with more limited studies showing no difference in MDSCs by HPV status (47, 49–54). Using single-cell RNA sequencing, Cillo and colleagues found HPV-positive tumors to be enriched in CD4 conversion cells with different differentiation trajectories and have increased germinal center B cells with increased ligand/receptor interactions between these B cells and T follicular helper cells, compared to HPV negative (48). Other studies have also shown an increase in B cells and that the B cells or more activated in HPV-positive tumors (49, 55, 56). HPV-positive HNSCC was observed to have a higher percentage of tumors with TCIP high phenotype compared to negative, with 51 *vs.* 21% TCIP high, respectively. Dividing HNSCC into previously established molecular subtypes atypical, basal, classical, and mesenchymal, the atypical and mesenchymal subtypes had the highest degree of immune infiltration and activity, with HPV-positive HNSCC making up the majority of the atypical subtype, while the classical subtype, which most resembles SCC of the lung, had the lowest (46).

Tobacco remains a risk factor for HNSCC, and multiple studies have found an increased molecular smoking signature is associated with a significantly higher mutational burden but lower immune infiltration and activation, independent of HPV status, and was a stronger predictor than reported smoking history. The smoking signature was higher in p53 mutated patients and larynx primary site (46, 57). Interestingly, the opposite was seen in SCC of the lung where higher smoking signature was associated with increased immune infiltration, potentially driven by increased inflammatory response in the lungs compared to the mucosa of the head and neck (57). Various mutations have correlated with the TIME including p53 mutations, deletion of chromosome 3p, deletion of CDKN2a, as well as NSD1, TGIF1, and EGFR mutations associated with lower immune infiltration/activation status, whereas mutation in CASP8, NSD1, HRAS, EP300, and EPHA2 has been associated with increased immune infiltration and activation (46, 47, 57). Increased intratumoral hypoxia has been associated with a more immune-suppressed TIME in HNSCC (58, 59).

Taken together, HNSCC while heterogeneous is generally associated with a TIME that may be infiltrated with immune cells but also one in which immune regulatory mechanisms are abundant. Amongst HNSCC, including HPV positive, there is a range of immune infiltration and activation status. Numerous studies have looked at the prognostic implications of various features of the TIME including cellular populations and co-signaling molecule expression, with overall conflicting data on prognostic implications, likely owing the limitations of looking at static isolated features amongst a dynamic immune response (49). It should be noted that most of our studies in HNSCC that guide our understanding of the TIME, including the important genomic and transcriptomic studies highlighted above, analyzed tumors mostly from the upfront locally advanced setting. Data are significantly limited on the changes in the TIME at recurrence after curative intent therapy. Waterman et al. uniquely compared paired primary and recurrent tumors and found a significant decrease in B cells, CD8 T cells, and NK cells, and a downward trend in CD8/Treg ratio in recurrent tumors. Additionally, receipt of adjuvant chemoradiation was associated with a significant decrease in B cells and a greater decrease in CD8/Treg ratio, an increase in macrophages and neutrophils of myeloid lineage, as well as downregulation of genes associated with cytokines and B cell immune response (60). Thus, the tumor microenvironment of recurrent/metastatic patients likely represents a more immune-suppressed phenotype compared to initial presentation.

Immunotherapy seeks to reverse the tumor-driven evasion and downregulation and use the immune system to eradicate cancer. In HNSCC, like other solid tumors, this has mostly been in the form of agents targeting co-signaling molecules, especially the PD-1:PD-L1 pathway which have changed standard of care. However, there is also ongoing evaluation of other checkpoint inhibitors, vaccines, as well as T cell therapy and additionally how chemotherapy and radiation can enhance immunotherapy.

## Immunotherapy as Standard of Care in R/M HNSCC

Prior to immunotherapy, platinum-based chemotherapy with or without cetuximab had been the standard systemic treatment for R/M HNSCC for over a decade (61). Unfortunately, the prognosis of patients receiving chemotherapy was poor, especially in the platinum failure setting (62). After promising efficacy of anti-PD-1/PD-L1 mAbs in smaller single-arm trials, randomized phase III trials first in the platinum failure setting and then in the frontline setting were conducted and have changed the standard of care systemic treatment for R/M HNSCC patients. Results of these phase III trials are summarized in **Table 1**.

**Table 1:** Completed Phase III studies of anti-PD-1/PD-L1 mAb therapy in Recurrent/Metastatic HNSCC.

Study	Study agents	Setting		ORR^1^	OS, HR, (95% CI), P value^2^	PFS, HR, 95% CI, P value^3^	FDA approval
CHECKMATE 141	(1) Nivolumab (2) CONTROL: MTX, Docetaxel, or Cetuximab	Platinum Failure		(1) 13.3% (2) 5.8%	(1) 7.5, HR 0.70 (0.51, 0.96), p:0.01 (2) 5.1, reference	(1) 2.0, HR 0.89 (0.70, 1.13) (2) 2.3, reference	Platinum Failure
KEYNOTE 040	(1) Pembrolizumab (2) CONTROL: MTX, Docetaxel, or Cetuximab	Platinum Failure		(1) 14.6% (2) 10.1%	(1) 8.4, HR 0.80 (0.65, 0.98), p:0.0161 (2) 6.9, reference	(1) 2.1, HR 0.96 (0.79, 1.16) (2) 2.3, reference	Platinum Failure
EAGLE	(1) Durvalumab (2) Durvalumab + Tremelimumab (3) CONTROL: MTX, Taxane, Cetuximab, 5-FU, Capecitabine, TS-1	Platinum Failure		(1) 17.9% (2) 18.2% (3) 17.3%	(1) 7.6, HR 0.88 (0.72, 1.08) (2) 6.5, HR 1.04 (0.85, 1.26) (3) 8.3, reference	(1) 2.1, HR 1.02 (0.84, 1.25) (2) 2.0, HR 1.09 (0.90, 1.33) (3) 3.7, reference	No
KEYNOTE 048	(1) Pembrolizumab (2) Pembrolizumab + Platinum + 5-FU (3) CONTROL: Cetuximab + Platinum + 5-FU	First line	Total population	(1) 17% (2) 36% (3) 36%	(1) 11.6, HR 0.85 (0.71, 1.03) (2) 13.0, HR 0.77 (0.63, 0.93), p:0.0034 (3) 10.7, reference	(1) 2.3, HR 1.34 (1.13, 1.59) (2) 4.9, HR 0.92 (0.77, 1.10) (3) 5.2, reference	First Line Treatment 1. Pembrolizumab plus platinum/5-FU for all patients 2. Pembrolizumab monotherapy for CPS ≥1
			PD-L1 CPS ≥1	(1) 19% (2) 36% (3) 36%	(1) 12.3, HR 0.78 (0.64, 0.96), p:0.0086 (2) 13.6, HR 0.65 (0.53, 0.80), p:<0.0001 (3) 10.4, reference	(1) 3.2, HR 1.16 (0.96, 1.39) (2) 5.0, HR 0.82 (0.67, 1.00) (3) 5.0, reference	
			PD-L1 CPS ≥20	(1) 31% (2) 54% (3) 44%	(1) 14.8, HR 0.58 (0.44, 0.78), p:0.0007 (2) 14.7, HR 0.60 (0.45, 0.82), p:0.0004 (3) 11.0, reference	(1) 3.4, HR 0.99 (0.76, 1.29) (2) 5.8, HR 0.76 (0.58, 1.01) (3) 5.3, reference	
			PD-L1 CPS <1^4^	(1) 2% (2) 12% (3) 19%	(1) 7.9, HR 1.51 (0.96, 2.37) (2) 11.3, HR 1.21 (0.76, 1.94) (3) 11.3, reference	(1) 2.1, HR 4.31 (2.63, 7.08) (2) 4.7, HR 1.46 (0.93, 2.30) (3) 6.2, reference	
			PD-L1 CPS 1-19^4^	(1) 18% (2) 34% (3) 45%	(1) 10.8, HR 0.86 (0.66, 1.12) (2) 12.7, HR 0.71 (0.54, 0.94) (3) 10.1, reference	(1) 2.2, HR 1.25 (0.96, 1.61) (2) 4.9, HR 0.93 (0.71, 1.21) (3) 4.9, reference	
JUNIPER-02^5^	(1) Cisplatin + Gemcitabine + Camrelizumab (2) Cisplatin + Gemcitabine + Placebo	First Line		(1) 88% (2) 81%	(1) Not Reached, HR 0.67 (0.41,1.11) (2) 22.6, reference	(1) 10.8,HR 0.51 (0.37,0.69), p<0.001 (2) 6.9, reference	Pending

^1^Overall Response Rate.

^2^Overall survival in months (median), Hazard ratio, 95% Confidence interval, P value shown if significant.

^3^Progression-free survival in months (median), Hazard ratio, 95% Confidence interval, P value shown if significant.

^4^Data retrieved from exploratory post-hoc analysis of KEYNOTE 048, p values are not applicable.

^5^JUNIPER-02 included nasopharyngeal carcinoma only. All other studies listed included squamous cell carcinoma of the oral cavity, oropharynx, larynx, hypopharynx.

MTX, Methotrexate; 5-FU, 5-Fluorouracil; CPS, Combined Positive Score.

Nivolumab and Pembrolizumab, both IgG4 anti-PD-1 monoclonal antibodies, were evaluated in phase III trials in R/M HNSCC patients with oral cavity, oropharynx, larynx, or hypopharynx primary after failure of platinum-based chemotherapy, and compared to investigator choice chemotherapy (Docetaxel, Cetuximab, or Methotrexate). Platinum failure was defined as progression within 6 months of platinum-based chemotherapy for R/M disease or within 6 months of platinum-based chemoradiation given in the curative intent setting. CHECKMATE-141 was the first phase III clinical trial to report efficacy and demonstrated significantly longer OS with nivolumab compared to chemotherapy (hazard ratio [HR] for death 0.70, 95% CI [0.51, 0.96], p=0.01). Importantly, nivolumab was better tolerated (G3/4 AEs 13.1 *vs.* 35.1% for nivolumab *vs.* chemotherapy respectively) and improved quality of life (63, 64). With these positive results, Nivolumab became the first therapeutic to significantly improve overall survival in R/M HNSCC patients that had failed platinum-based chemotherapy (63). In KEYNOTE 040, a similarly designed trial, pembrolizumab also improved overall survival compared to chemotherapy (65). Notably, both trials did not require PD-L1 expression for entry, and the primary endpoint was not powered by PD-L1 status. Neither study showed a significant difference in progression-free survival (PFS). Similar to other solid tumors, prolongation of overall survival but not PFS was likely driven by most patients on anti-PD-1 mAb progressing at first imaging evaluation, with the durability of the therapeutic effect for responders, and also a proportion of those with stable disease, driving the OS benefit. For example, while only 13% had a response with Nivolumab, the duration of response was a median of 9.7 months (2.8 to 32.8+), more than double that of chemotherapy (66). Based on the findings of CHECKMATE-141 and KEYNOTE-040, the FDA approved nivolumab and pembrolizumab monotherapy in patients with R/M HNSCC who had failed platinum-based chemotherapy in 2016.

Anti-PD-L1 mAbs as monotherapy and in combination with anti-CTLA4 mAb have also been evaluated in the platinum failure setting. After initial phase II trials with durvalumab in PD-L1 high (HAWK) and durvalumab, durvalumab plus tremelimumab, or tremelimumab alone in PD-L1 low patients (CONDOR), the phase III EAGLE trial was initiated and randomized platinum failure R/M HNSCC patients to durvalumab plus tremelimumab, durvalumab monotherapy, or investigator choice standard of care chemotherapy. This trial was dually powered for OS comparison of durvalumab and combination durvalumab plus tremelimumab separately, compared to chemotherapy. There was no difference in OS with durvalumab (HR 0.88, 95% CI [0.72, 1.08], p=0.20) or durvalumab plus tremelimumab. (HR 1.04, 95% CI [0.85, 1.26], p=0.76) compared to chemotherapy. Accepting the limitations of cross-trial comparisons, it is notable that while the median OS with durvalumab was similar to nivolumab in Checkmate 141 (7.6 *vs.* 7.5 months, respectively), the median OS of the control arm was numerically longer in EAGLE compared to CHECKMATE 141 (8.3 months *vs.* 5.1 months respectively). Exploratory analysis from EAGLE suggests that this higher-than-expected OS in the control group may have come from imbalance in baseline characteristics (higher percentage of ECOG PS 0 and distant metastasis only in the control arm), increased usage of paclitaxel in the control arm, which was not a choice in CHECKMATE 141 or KEYNOTE 040, and subsequent receipt of anti-PD-1 mAb therapy (67). Notably, there are differences between anti-PD-1 and PD-L1 mAbs. Both block the interaction of PD-1:PD-L1, but anti-PD-L1 mAb’s block the interaction of PD-L1:CD80, whereas anti-PD-1 mAbs inhibit the ligation of PD-1 by PD-L2. However, whether this difference has a clinically relevant effect is not known.

Success in the platinum failure setting led to evaluation of immunotherapy in the frontline systemic treatment of R/M HNSCC patients. The phase III randomized trial KEYNOTE-048 evaluated pembrolizumab monotherapy and platinum/5FU/pembrolizumab each separately compared to the EXTREME regimen (platinum/5FU/cetuximab) for the total population, PD-L1 combined positive score (CPS) ≥1, and ≥ 20 (**Table 1**). CPS was defined as the number of PD-L1–positive cells [tumor cells, lymphocytes, macrophages] divided by the total number of tumor cells × 100. Pembrolizumab monotherapy significantly improved OS in patients with CPS ≥1 and ≥20. While the response rate was lower than chemotherapy (19–21 *vs.* 36%, respectively), the median duration of response with pembrolizumab monotherapy was fivefold higher (median 20.9 *vs.* 4.5 months, respectively). Chemotherapy plus pembrolizumab significantly improved OS for all three populations. There was no significant difference in response rate and PFS between chemotherapy plus pembrolizumab and the EXTREME regimen. As expected, pembrolizumab monotherapy was associated with less toxicity, while a similar rate of adverse events occurred with platinum/5FU/pembrolizumab as compared to EXTREME (68). This led to the FDA approval in 2019 of platinum/5FU plus pembrolizumab for all patients and pembrolizumab monotherapy only for patients with a PD-L1 CPS ≥1. The phase III KESTREL trial randomized patients 2:1:1 to durvalumab alone, durvalumab plus tremelimumab, and the EXTREME regimen. The primary endpoint was OS for durvalumab monotherapy *vs.* EXTREME in PD-L1 high expressers (tumor cell expression of >50% or tumor-infiltrating lymphocyte expression >25%) and secondary endpoint of OS for durvalumab plus tremelimumab *vs.* EXTREME for all patients. While the data are not available yet from the trial, by press release it was announced that the trial had failed to meet these endpoints.

KEYNOTE 048 importantly represents the first change in frontline therapy since the EXTREME regimen in 2009; however, questions that effect day-to-day practice remain. One is whether patients with a CPS ≥20, which represented 44% of PD-L1 expressers, drove the benefit with pembrolizumab monotherapy in the CPS ≥1 group. Put another way, is pembrolizumab monotherapy enough for a patient with a PD-L1 CPS 1-19? Exploratory subgroup analysis from KEYNOTE 048 showed there was still a benefit from pembrolizumab compared to EXTREME for CPS 1-19 (HR 0.86 95% CI [0.66–1.12]) albeit less benefit relative to CPS ≥20 patients (HR 0.58 95% CI (0.44–0.78)) (69). In practice, the decision to choose pembrolizumab monotherapy *versus* chemotherapy plus pembrolizumab for a patient with CPS 1-19 depends on multiple patient and disease factors, such as tumor and symptom burden, comorbidity, and performance status. In patients with a PD-L1 CPS 1-19 with high tumor burden and/or significant symptoms that can tolerate chemotherapy, we favor chemotherapy plus pembrolizumab as a standard of care treatment, to maximize potential response, which can translate directly into a quality-of-life benefit. Additionally, the total population is not the same as PD-L1 negative patients, which accounted for only 15% of the patients in the trial. In practice most providers will know if a patient has a PD-L1 CPS <1. Subgroup analysis for PD-L1 negative patients treated with chemotherapy plus pembrolizumab favored the EXTREME regimen with a HR of 1.22 (95% CI [0.76–1.94]) (69); however, this should not affect practice given very small patient numbers in this cohort, and chemotherapy plus pembrolizumab is still the new standard of care for a patient with known PD-L1 negative status.

In summary, current frontline standard of care systemic therapy options for R/M HNSCC of the oral cavity, oropharynx, larynx, and hypopharynx include pembrolizumab monotherapy or platinum/5FU plus pembrolizumab for PD-L1 expressers by CPS or platinum/5FU plus pembrolizumab for all patients. While this change in frontline systemic therapy has limited the applicability of nivolumab and pembrolizumab monotherapy for platinum failure patients, it is notable that patients that fail platinum-based chemoradiation within 6 months still meet criteria for anti-PD-1 monotherapy regardless of PD-L1 status.

Owing to some differences in biology and higher risk of distant metastasis, nasopharyngeal carcinoma has been evaluated in trials separately from other HNSCC sites. Both Pembrolizumab and Nivolumab were evaluated in single-arm phase II trials with treatment with single agent nivolumab in the platinum failure setting associated with an RR of 20.5% with a median PFS and OS of 2.8 months and 17.1 months, respectively, in the 44 patients enrolled in the trial (70, 71). These trials led to a category 2B NCCN recommendation for pembrolizumab and nivolumab as an option after failure of first-line Cis/Gem, as a randomized phase III trial will not be conducted in the platinum failure setting for nasopharyngeal carcinoma. Combination Ipilimumab and Nivolumab was studied in 40 patients with EBV-positive nasopharyngeal carcinoma with an RR of 35% and median PFS and OS of 5.3 and 17.6 months, respectively (72). This compares favorably to an RR of 18% observed with combination anti-CTLA4 plus anti-PD-L1 in non-nasopharyngeal HNSCC (67). The first phase III randomized trial in the frontline setting, JUPITER-02, was presented at the ASCO 2021 annual meeting. This trial randomized patients to Cisplatin plus Gemcitabine plus anti-PD-1 mAb camrelizumab *vs.* Cisplatin plus Gemcitabine plus placebo. The study met its primary endpoint of PFS with a significant improvement in PFS with the addition of camrelizumab with a median PFS of 10.8 months *vs.* 6.9 months in the control arm (HR 0.51[95% CI 0.37 to 0.69], P<0.0001). Notably, 82% of patients had undifferentiated carcinoma, and patients were enrolled regardless of PD-L1 status without stratification. It is expected that this will be a practice-changing trial, and this new regimen has received breakthrough therapy designation by the FDA.

## Predictive Biomarkers

While the approvals of Pembrolizumab and Nivolumab have been a great stride for the field, only a minority of patients benefit from blockade of the PD-1:PD-L1 pathway. As such there continues to be a need for predictive biomarkers for efficacy. Biomarkers evaluated in R/M HNSCC include PD-L1, immune gene expression, tumor mutational burden, as well as the effect of viral etiologies such as HPV.

By far the most vetted biomarker across solid tumors and in R/M HNSCC is PD-L1 expression with higher expression predictive of increased efficacy. In Checkmate 141, using a cut point of ≥1% tumor membranous PD-L1 expression, there was a greater reduction in the risk of death with Nivolumab *versus* standard therapy in positive patients (HR for death: 0.55; 95% CI: 0.36–0.83) compared to PD-L1 negative (HR for death: 0.89; 95% CI: 0.54–1.45). Updated analysis after extended follow-up showed that the benefit of Nivolumab in PD-L1 negative patients increased over time, with a reduction in the HR for death to 0.73, while benefit was maintained in PD-L1 expressing patients (63, 66). The addition of PD-L1 expression on tumor-infiltrating lymphocytes (TIL) has shown better predictive value compared to tumor PD-L1 expression alone in HNSCC. For example, in a retrospective analysis of patients treated with pembrolizumab, there was no significant difference in response by tumor PD-L1 expression alone (defined as ≥1%), but when combination tumor plus TIL PD-L1 expression was used, PD-L1 positive HNSCC patients had a significantly higher RR, PFS, and OS (73). Added predictive value with inclusion of TIL PD-L1 was also shown in exploratory subgroup analysis of checkmate 141 and Keynote 048 (68, 74).

Immune gene expression profiles (GEP) have also shown predictive value with anti-PD-1 mAb treatment (75–78). For example, in HNSCC, a composite score based on six Interferon gamma related genes (CXCL9, CXCL10, IDO1, IFNG, HLA-DRA, and STAT1) was predictive of response and PFS with pembrolizumab. It showed a high negative predictive value (95%) as only 5% of patients below the Youden index had a response compared to 40% with a score above (77). First observed in melanoma, higher tumor mutational burden (TMB) has also been associated with increased efficacy with anti-PD-1 mAb therapy in R/M HNSCC patients (79, 80). Other new potential biomarkers include intratumoral hypoxia, which has been associated with immunosuppression. Evaluation of anti-PD-1 treated R/M HNSCC patients showed lower intratumoral hypoxia was associated with increased efficacy and was independently associated with clinical benefit rate and PFS in multivariate analysis, which included tumor infiltrating CD8 T cells, the latter of which has also shown predictive value with anti-PD-1 mAb therapy (81, 82). Hypoxia as a biomarker is promising because it also has the potential to be modulated by therapeutics. While the oral microbiome was not predictive in HNSCC patients treated with Nivolumab, the intestinal microbiome has not been evaluated to date (83).

In HNSCC, there are two relevant viral etiologies, EBV for nasopharyngeal carcinoma and HPV for oropharyngeal. While HPV-positive oropharyngeal SCC is associated with a better prognosis in the R/M setting, the magnitude is much less as compared to the locally advanced setting, and systemic therapy alone is still only palliative (84). HPV-positive oropharyngeal SCC has made up approximately 20% of patients enrolled in anti-PD-1/L1-based trials, with conflicting data on whether HPV status is associated with increased efficacy with these agents. Subgroup analysis of Checkmate 141 showed a similar magnitude of OS benefit with nivolumab compared to chemotherapy in HPV-positive oropharyngeal cancer (9.1 *vs.* 4.4 months, HR 0.60; 95% CI, 0.37–0.97) *versus* negative (7.7 *vs.* 6.5 months, HR 0.59; 95% CI 0.38–0.92). However, this comparison of the difference in efficacy relative to chemotherapy within the same group is different than the question as to whether an HPV-positive patient is more likely to respond and have better efficacy from anti-PD-1/L1 mAb blockade compared to an HPV-negative patient. Analysis of the tumor microenvironment shows a spectrum of T cell activation status in both HPV-positive and HPV-negative patients, with a higher percentage of a T cell inflamed phenotype in HPV-positive patients, 51 *vs.* 21%, respectively (54). When compared by HPV status directly, some analysis show higher efficacy and some no difference. These analyses are challenged however by low sample sizes and also lack of controlling for PD-L1 status. For example, analysis of patients from Keynote 055, which included both PD-L1-positive and -negative patients, showed similar response rates, while analysis of the HAWK trial and Keynote 012, both of which included only PD-L1-positive patients, showed increased response rate and OS for HPV-positive oropharyngeal compared to HPV negative (77, 85, 86). While data are somewhat conflicting, what is clear is that both HPV-positive and HPV-negative patients benefit from anti-PD-1/L1 mAb therapy. Outside of the oropharynx there is not a defined role for HPV in oncogenesis or prognosis. However, interestingly, in the phase II HAWK study, both HPV-positive oropharyngeal and HPV-positive non-oropharyngeal patients had similarly higher efficacy with durvalumab compared to HPV negative. This suggests that perhaps the effect of HPV on the tumor microenvironment even as a bystander in non-oropharyngeal SCC may be associated with increased efficacy (86). However, this needs to be validated before any conclusions can be made.

Less is known about the predictive value of EBV as most reported prospective trials have included only EBV-positive patients. Reduction of plasma EBV DNA after initiation of nivolumab showed some trend in responders but was not significantly associated with efficacy in a small subgroup (70). A small retrospective analysis showed a numerically higher RR in EBV positive compared to negative, but it was similarly not statistically significant (87).

The interaction of PD-L1, HPV, GEP, and TMB has been analyzed in HNSCC. In 258 R/M HNSCC patients treated with pembrolizumab, response and PFS were significantly associated with TMB, GEP, and PD-L1 expression, as well as OS for the latter two biomarkers. Amongst HPV-positive patients, there was a suggestion that TMB was less predictive compared to GEP and PD-L1 (80). The reason for this may be that viral etiology was enough for immune activation, resulting in less dependence on a higher number of mutations and resulting increased neoantigens to drive immune recognition. While there was moderate correlation between PD-L1 and GEP, TMB did not correlate with either. TMB, GEP, and PD-L1 were independently associated with response, with those with high TMB and PD-L1 or high GEP and high TMB having the greatest likelihood of response (34%) (80). This important analysis highlights that even with two favorable biomarkers, the response rate was still only 34%, speaking to the complexity of the immune microenvironment. However, an unfavorable combination of these biomarkers was associated with a high negative predictive value.

While the predictive value of these biomarkers is not absolute, a high negative predictive value for anti-PD-1 monotherapy is important, especially in the frontline setting when considering adding chemotherapy or recommending clinical trial. Another important question is how much the tumor immune microenvironment changes over time in an individual patient including after various therapeutic interventions. The aforementioned analysis all include archival tissue of various durations as well as some patients with a new biopsy before treatment. There is direct data that the predictive value of PD-L1, for example, is similar in archival *vs.* tissue samples immediately prior to anti-PD-L1 treatment, and lack of a significant change in PD-L1 expression in paired primary and recurrent tumors (60, 88). This brings up the question as to whether a patient’s immune phenotype and thus likelihood of efficacy is relatively fixed over their treatment course. Further analysis of changes over time in these biomarkers are needed.

## Future Directions

The success of Checkmate 141, Keynote 040, and more recently Keynote 048 represents great progress for patients with R/M HNSCC. With progress comes important new questions and goals in order to continue to improve outcomes. This includes the integration of immunotherapy earlier in the recurrent setting with salvage surgery and/or reirradiation, improving efficacy in the frontline setting and the role of immunotherapy after failure of anti-PD-1 mAb-based therapy. Ongoing trials for each of these categories are shown in **Table 2**.

**Table 2:** Ongoing immunotherapy trials in Recurrent/Metastatic HNSCC*.

NCT	Trial name	Phase	Experimental Arm	Control Arm	Primary Endpoint
**Adjuvant immunotherapy after surgical resection of recurrent HNSCC**					
04671667	ECOG 3191	II	1. Adjuvant Reirradiation plus Pembrolizumab 2. Adjuvant Pembrolizumab monotherapy	Reirradiation plus platinum	Overall Survival
03355560		II	Neoadjuvant and adjuvant Nivolumab plus Lirilumab		Disease-Free Survival
03406247		II	1. Adjuvant Nivolumab 2. Adjuvant Nivolumab plus Ipilimumab		Disease-Free Survival
03355560		II	Adjuvant Nivolumab		Toxicity
02769520		II	Adjuvant Pembrolizumab		Disease-Free Survival
**Reirradiation plus immunotherapy**					
03546582	KEYSTROKE	II	SBRT reirradiation plus Pembrolizumab	SBRT alone	Progression-Free Survival
02289209		II	Hyperfractionated reirradiation plus Pembrolizumab		Progression-Free Survival
03521570		II	Re-irradiation plus Nivolumab (definitive or adjuvant)		Progression-Free Survival
03803774		I	BAY1895344 plus SBRT and Pembrolizumab		Toxicity
**Frontline Systemic Therapy Trials (PD-L1 positive)**					
**Combination immunotherapy**					
04634825		II	Enoblituzumab plus Retifanlimab		Response Rate
04633278		II	CMP-001 plus pembrolizumab		Response Rate
04260126	VERSATILE002	II	PDS0101 plus pembrolizumab		Response Rate
04398524		II	Cemiplimab plus ISA101b	Cempilimab + Placebo	Response Rate
04034225		I/II	SNS-301 Intra-tumor injection + Pembrolizumab		Toxicity
04453046		I	Hemopurifier plus pembrolizumab		Safety
04408898	SPEARHEAD 2	II	ADP-A2M4 plus pembrolizumab		Response Rate
**Molecularly targeted therapy plus immunotherapy**					
04199104	LEAP-010	III	Pembrolizumab plus Lenvatinib	Pembrolizumab plus Placebo	Overall Survival, Response Rate, Progression-Free Survival
04114136		II	1. Metformin plus Pembrolizumab 2. Rosiglitazone plus Pembrolizumab	Pembrolizumab	Response Rate
**Frontline Systemic Therapy Trials (regardless of PD-L1 status)**					
**Combination immunotherapy**					
02741570	Checkmate 651	III	Ipilimumab plus Nivolumab	EXTREME	Overall Survival
04634825		II	Enoblituzumab plus tebotelimab (PD-L1 negative)		Response rate
**Molecularly targeted therapy plus immunotherapy**					
03468218		II	Pembrolizumab plus Cabozantinib		Response Rate
03498378		I	Avelumab plus Cetuximab plus Palbociclib		Toxicity
**Cytotoxic chemotherapy plus immunotherapy**					
04489888	KEYNOTE B10	IV	Pembrolizumab plus Platinum plus Paclitaxel		ORR
04282109	NIVOTAX	II	Paclitaxel plus Nivolumab	Paclitaxel plus cetuximab	Overall Survival
**Immunotherapy failure trials**					
**Immunotherapy Combination**					
04590963	INTERLINK-1	III	Monalizumab + Cetuximab	Placebo + Cetuximab	Overall survival
04326257		II	1. Nivolumab plus Relatlimab 2. Nivolumab plus Ipilimumab		Response Rate
04150900		II	Pembrolizumab plus Bavituximab		Response Rate
04408898		II	ADP-A2M4 T cells plus pembrolizumab		Response Rate
03769467		I/II	Tabelecleucel plus pembrolizumab		Toxicity/Response Rate
04196283		I	1.ABBV-368 plus Tilsotolimod plus Nab-paclitaxel plus ABBV-181 2. ABBV-368 plus Tilsotolimod plus Nab-paclitaxel 3. ABBV-368 plus Tilsotolimod		Toxicity
**Molecular targeted therapy plus Immunotherapy**					
04428151	LEAP-009	II	Pembrolizumab plus Lenvatinib	1. Chemotherapy (Taxane, cetuximab, or capecitabine) 2. Lenvatinib	Response Rate
03019003		I/II	Decitabine plus Durvalumab		Toxicity/Response Rate
04624113	–	I/II	Tazemetostat plus Pembrolizumab		Toxicity/Response Rate

*Trials included are those that are focused entirely in HNSCC.

While salvage resection is generally considered the most aggressive option for locoregionally recurrent HNSCC, long-term survival is still poor (89). Similarly, there is a need for improvement in outcomes with reirradiation plus concurrent chemotherapy, including with reduced toxicity. Preclinical data suggest radiation has pro-immunogenic as well as immunosuppressive effects (90), and it will take clinical trials to best determine how to maximize the former in patients. Trials combining reirradiation and immunotherapy in the recurrent setting are ongoing. A phase II trial with hyperfractionated reirradiation (1.2 Gy twice daily for a total of 60 Gy) plus pembrolizumab for patients with locoregional recurrence without a surgical option, was first to report acute toxicity, without unexpected adverse events, with the trial now accruing towards its primary endpoint of PFS (NCT02289209) (91). The NRG foundation Keystroke trial is ongoing in the same setting comparing reirradiation with SBRT alone *vs.* SBRT plus pembrolizumab (NCT03546582). Nivolumab is being combined with daily radiation in another single-arm phase II trial that includes both definitive and adjuvant reirradiation patients (NCT03521570). While a randomized phase II trial showed adjuvant reirradiation plus concurrent chemotherapy significantly improved DFS post-salvage resection, there was no difference in OS, providing clinical equipoise for challenge, including with the evaluation of immunotherapy alone in the adjuvant setting (92). Multiple smaller studies are evaluating anti-PD-1 mAb monotherapy after salvage resection. One such single-arm trial reported a pre-planned interim analysis at ESMO 2019 passing its futility boundary for efficacy with estimated DFS of 55% at 10.2 months, and continues on towards its primary endpoint (93). An ECOG trial has recently opened for patients that undergo salvage resection of recurrent or second primary HNSCC that have high-risk features of ENE and/or positive margins and PD-L1 CPS ≥1. Patients in this trial are randomized to pembrolizumab monotherapy for 12 months, reirradiation (2 Gy daily to total 60 Gy) plus pembrolizumab for 12 months, or control arm of reirradiation plus concurrent weekly platinum chemotherapy. Both experimental arms are being compared to control separately with a primary endpoint of OS (NCT04671667). This trial enriches for those more likely to benefit from anti-PD-1 mAb monotherapy and in combination with radiation by including only PD-L1 expressers. Notably, PD-L1-positive patients were the only subgroup that benefited from the addition of avelumab to chemoradiation in exploratory analysis of the Javelin trial in the definitive locally advanced setting (94).

The FDA approval of frontline pembrolizumab alone and in combination with chemotherapy has driven new trials trying to build upon this new standard of care. This has come in the form of combination immunotherapy, molecularly targeted therapy plus immunotherapy, and additional combinations of cytotoxic chemotherapy plus immunotherapy. One of the key questions is whether we can increase the efficacy of pembrolizumab monotherapy with another immunotherapy or targeted agent and avoid the added toxicity from cytotoxic chemotherapy. GSK3359609 is an inducible T cell co-stimulatory receptor (ICOS) agonist. ICOS is a member of the CD28 co-receptor family. Preliminary data with GSK3359609 plus pembrolizumab in immunotherapy naïve patients, 53% of which had received at least one prior line of therapy, showed an RR of 26% with four complete responses. The median PFS and OS of 4.2 months and 13.1 months respectively. This led to a phase II/III trial of Pembrolizumab +/− GSK3359609 in the frontline setting in patients with PD-L1 expression; however, after a planned interim analysis of efficacy, the decision was made to not transition to the phase III component (95). Promising efficacy with anti-B7H3 mAb Enoblituzumab plus pembrolizumab with a response rate of 33% in platinum failure anti-PD-1 naive HNSCC patients has led to a phase II study with enoblituzumab plus anti-PD-1 retifanlimab in PD-L1-expressing patients (96). While the phase III Kestrel trial was reported as negative, fully accrued is Checkmate 651 evaluating Ipilimumab plus Nivolumab in the frontline setting *versus* EXTREME, and we await these results. Specifically, in HPV-positive oropharyngeal SCC, based on an RR of 33% with combination nivolumab and ISA 101, a synthetic long-peptide HPV-16 vaccine, a randomized phase II trial is ongoing including frontline and platinum failure patients (NCT03669718). Given the morbidity and mortality driven by local disease in HNSCC, immunotherapy injected directly into the tumor could be a potentially clinically meaningful option for the subset of patients with accessible lesions. Early data on stimulator of interferon genes (STING) agonist ADU100 plus pembrolizumab in the frontline PD-L1-expressing setting showed tolerability and PR in 5/8 patients (97). Promising data with TLR9 agonist CMP001 in melanoma has led to the exploration of this agent plus pembrolizumab also in the frontline R/M HNSCC setting (NCT04633278) (98).

In terms of combination therapies targeting molecular pathways, LEAP-010 is a phase III placebo-controlled, randomized study of Pembrolizumab with or without Lenvatinib as first-line therapy in PD-L1-expressing patients. Additionally, promising efficacy has been observed with IgG1 mAb cetuximab plus anti-PD-1 mAb. For example, cetuximab plus pembrolizumab in immunotherapy naïve patients showed an RR of 45% with a median duration of response of 14.9 months (99). Different chemotherapy backbones are also being evaluated, as well as adding additional immunotherapy to chemotherapy. For example, KEYNOTE B10 is an ongoing study of Pembrolizumab with Carboplatin and Paclitaxel as first-line treatment for R/M HNSCC (NCT04489888).

Driven by all R/M HNSCC patients now receiving anti-PD-1 mAb-based therapy in the frontline setting, there is great and growing need for better therapeutics after anti-PD-1 failure. The majority of immunotherapy-based trials are in early phase with most combinations being tested in phase I trials with expansion cohorts, some of which include HNSCC. Preliminary data have been reported for two cetuximab-based combinations. Cetuximab plus nivolumab showed RR of 17% and SD in an additional 17% in 23 patients that had failed prior anti-PD-1 mAb therapy (100). Natural Killer Group 2A (NKG2A) inhibitor Monalizumab plus cetuximab was associated with an RR of 20%, SD in 37.5%, and median duration of response of 5.2 months (95% CI; 3.9-not reached). This combination is currently being compared to cetuximab alone in a phase III clinical trial for patients that have failed prior anti-PD-1 and platinum (NCT04590963) (101). Additionally, Lenvatinib plus pembrolizumab is also being tested in the anti-PD-1 failure setting compared to standard of care chemo and Lenvatinib monotherapy in a randomized phase II Trial (NCT04428151).

Adoptive T cell therapy especially CAR T cells have shown significant efficacy in hematologic malignancies. In solid tumors, challenges to adoptive T cell therapy include appropriate antigen targets and adequate penetration into the tumor microenvironment. While headway has been made in some solid tumors such as melanoma, data in HNSCC are more preliminary with trials ongoing. Preliminary data using pan-ErbB targeted CAR-T cells showed tolerability and SD in 60% (3/5) at 6 weeks (102). Autologous TIL therapy Lifileucel in combination with pembrolizumab in anti-PD-1 naïve R/M HNSCC patients showed a response rate of 44% in nine patients with responses ongoing in three out of the four patients at a median follow up of 8.6 months (103). A trial with ADP-A2M4 targeting MAGE-A4-positive HNSCC in combination with pembrolizumab, also in anti-PD-1 naïve patients, is currently accruing. A number of studies have focused on viral antigens including EBV and HPV. Ten patients with R/M NPC positive for EBV encoded RNA and/or EBV-LMP1 refractory to multiple lines of therapy received autologous T cell therapy weekly ×4 doses then every 2–4 weeks. The clinical benefit rate was 60% with a PR in two patients and SD in four patients (104). Smith and colleagues reported a phase I trial with T cells generated by an adenovirus-based vector, AdE1-LMPpoly, which expands LMP1&2- and EBNA1-specific T cells also in EBV-positive advanced NPC. Out of 14 patients treated, SD was seen in 10 patients with a median time to progression of 66 days (range 38–420) (105). A larger phase II trial evaluated 35 patients treated with frontline carboplatin plus gemcitabine ×4 cycles followed by autologous EBV cytotoxic lymphocytes. While there was only minimally enhanced response beyond what is expected with chemotherapy alone, the median OS of 29 months compares favorably to the expected median OS of 22 months with platinum/gemcitabine alone (106). A phase III trial with this regimen is ongoing (NCT02578641). Tabelecleucel, an allogeneic T cell immunotherapy, is currently being evaluated with pembrolizumab in EBV positive NPC (NCT03769467). Small studies have evaluated targeting HPV E6 and E7. Twelve patients with HPV16-positive advanced cancer were treated with autologous genetically engineered T cells expressing a TCR against HPV16 E6. Two patients (anal SCC) had a PR, and the one oropharyngeal SCC patient experienced SD lasting 4 months (107). Another study evaluated targeting HPV16 E7 also with T cells with engineered TCR. This study included 12 patients, of which six patients achieved a PR and four SD. The study included four HNSCC patients, all of which had failed platinum and anti-PD-1. In the HNSCC patients there were two PRs and two SD with response/stability lasting for 3–4 months (108). While small sample sizes preclude conclusions, the higher efficacy in the latter study suggests that E7 may be a better target than E6 for HPV-positive patients. These trials highlight the feasibility of T cell therapy in HNSCC with larger trials needed to establish its efficacy. Similar to checkpoint inhibition, continued study of predictive biomarkers specifically for T cell therapy will be critical to guide selection of patients for this type of therapy.

With numerous frontline combination trials underway in patients with PD-L1 expression, we must strive not only for better efficacy but also concurrent knowledge on how to select the best therapy. This will be critically important if multiple new regimens improve OS in phase III trials. For example, meaningful to integration into everyday practice would be powering trials by CPS score subgroups so we would know whether a combination is effective in just CPS >20 or also CPS 1-19 patients. While only a select group of patients will have lesions amenable to intratumoral injection, consistent response of injected lesions could prove important in reducing at minimum morbidity. In addition to local effects, the key question is whether intratumoral injection in combination with anti-PD-1 will also enhance response in non-injected sites and ultimately improve mortality.

Important to making progress with combination cytotoxic chemotherapy in the immunotherapy era is a better understanding of the effect of chemotherapy on the tumor microenvironment. Preclinical data show immunogenic effects of numerous cytotoxic agents active in HNSCC such as Cisplatin, 5FU, and Taxane; however, there is suggestion that with repetitive doses, immunosuppressive effects can also occur (109, 110). While chemotherapy plus immunotherapy has improved OS in HNSCC and other solid tumors, the effect is additive at best. For example, in Keynote 048, while the RR was higher with chemotherapy plus pembrolizumab compared to pembrolizumab monotherapy (36 *vs.* 19% in PD-L1 expressers), the median duration of response was three times lower. One explanation for this is that the response in the additional 17% was driven by solely the chemotherapy with no benefit from the pembrolizumab. This highlights the need to examine the best type and sequence of cytotoxic chemotherapy and immunotherapy with the goal of achieving not only a higher response but a prolonged duration of response. Additionally, an open question is whether anti-PD-1 monotherapy should be continued after progression with subsequent addition of chemotherapy. A better understanding is also needed as to whether systemic agents can change a patient’s tumor microenvironment so that they may be more likely to benefit from immunotherapy with re-challenge either with anti-PD-1 again or novel combination immunotherapy.

With a seemingly infinite number of immunotherapy combination options being developed and tested, especially in the anti-PD-1 failure setting, we are searching for the next big step for the field. It is unlikely that any combination will work in 90% or even 50% of patients, but rather that a more personalized approach using a tumor microenvironment–driven selection strategy to choose the best combination may be the only way to get the majority of patients to benefit from immunotherapy. To do this we must start evaluation of selection strategies prospectively where clinical equipoise allows.

## Conclusion

Immunotherapy has transformed the field of oncology over the last decade including in head and neck cancer with a current standard of care role in the frontline and platinum failure setting in R/M HNSCC. It is an exciting time for both patients and providers with an explosion of new agents and clinical trials. While rare, it is amazing to see the durable benefit with anti-PD-1 mAb-based therapy achieved in some patients. But as a field we are also at a critical juncture as to how to take the next big leap after anti-PD-1 mAb therapy to help more patients benefit from immunotherapy. Undoubtedly, we will have to rein in our approaches focused and tailored by an increased understanding of the tumor immune microenvironment in patients, with the ultimate goal of a more personalized approach leading to benefit with immunotherapy in the majority of patients. While we have a lot more work to do, the future is brighter for our R/M HNSCC patients.

## Author Contributions 

All authors participated in the development, writing, and editing of the review article. All authors contributed to the article and approved the submitted version.

## Conflict of Interest

DZ received research support (institutional) for his role as principal investigator on trials with Merck, BMS, Macrogenics, Astrazeneca, Bicara, Lilly, Aduro, GSK, Checkmate pharmaceuticals. DZ served on an advisory board for Bluprint Medicines and Macrogenics.

The remaining authors declare that the research was conducted in the absence of any commercial or financial relationships that could be construed as a potential conflict of interest.

## Publisher’s Note

All claims expressed in this article are solely those of the authors and do not necessarily represent those of their affiliated organizations, or those of the publisher, the editors and the reviewers. Any product that may be evaluated in this article, or claim that may be made by its manufacturer, is not guaranteed or endorsed by the publisher.
